# Catheter-associated urinary tract infections in critical care: Understanding incidence, risk factors, and pathogenic causes in Palestine

**DOI:** 10.1371/journal.pone.0309755

**Published:** 2024-08-30

**Authors:** Razan Rabi, Ahmad Enaya, Dana Mufeed Jomaa, Mo’tasem Z. Dweekat, Shahd Raddad, Zain Tareq Saqfalhait, Dina Abu-Gaber

**Affiliations:** 1 Department of Internal Medicine, An-Najah National University Hospital, Nablus, Palestinian Territory; 2 Palestinian Ministry of Health, Jericho, Palestinian Territory; 3 Department of Internal Medicine, Palestinian Medical Complex, Ramallah, Palestinian Territory; University of Louisiana at Lafayette, UNITED STATES OF AMERICA

## Abstract

Catheter-associated urinary tract infections (CAUTI) are the most common secondary cause of bloodstream infection. CAUTI is particularly prevalent in critical care departments and developing countries, where the duration of catheterization remains the most significant risk factor. This study focused on the characteristics, risk factors, and outcomes of CAUTI patients in a tertiary care hospital setting. It also provides the incidence rate of CAUTI in an ICU setting in Palestine. The study adopted a retrospective observational design at a tertiary care hospital in Palestine. The data were collected from patient records as well as from nursing flow charts. Variables are reported as frequencies, percentages and means + standard deviations. Independent t-tests was used for numerical variables, while Pearson’s chi-square or Fisher’s exact test were used for categorical variables. Multivariate analysis was performed to adjust for confounders using binary logistic regression. Mortality risk factors were assessed using the proportional Cox regression model. Of the 377 patients included in the study, 33 (9%) developed CAUTI. Among CAUTI patients, 75% had Candida species isolated, with non-albicans Candida predominating (72%) fungal isolates. On the other hand, 25% of the patients had bacterial isolates in their urine, with a predominance of *Escherichia coli* growing in 36% of bacterial cultures. Multivariate regression analysis revealed that female gender, longer catheterization days, and corticosteroid use were associated with an increased risk of CAUTI. On the other hand, developing CAUTI, having a malignant disease, developing kidney injury, and developing shock were associated with increased mortality. This study highlighted the emerging presence of fungal and resistant bacterial CAUTI. It also emphasized that the risk of CAUTI was associated with a longer duration of urinary catheterization. The findings of this study may help formulate antimicrobial management and stewardship plans as well as emphasize the risk of urinary catheterizations.

## Introduction

Healthcare-associated infections (HCAIs) are infections acquired while receiving health care and are a major cause for concern because they are associated with increased mortality, morbidity, and healthcare costs. One of the most common HCAIs is catheter-associated urinary tract infections (CAUTI), which are infections of the urinary tract in individuals whose urinary tracts are catheterized or have been catheterized within the preceding 48 hours [1,2]. Moreover, CAUTIs are the most common secondary cause of bloodstream infections. The estimated cost of preventable CAUTI episodes is 115 million United States dollars (USD) to 1.8 billion USD [1].

According to the European Centre for Disease Prevention and Control (ECDC), approximately 98.4% of urinary tract infections (UTI) are related to the presence of a urinary catheter. The same report showed that a urinary catheter was used in 78% of the patient-days of all patients who stayed in the intensive care unit (ICU) for more than two days [3]. The duration of catheterization is the most important risk factor for developing CAUTI [4]. Furthermore, nosocomial infections are reportedly 3–5 times more frequent in the ICU than in other hospital departments. In addition, ICU-related CAUTI is associated with longer hospital stays, greater financial burden and greater antibiotic use [5]. The pooled prevalence of HCAI in different patient populations from 1995–2010 was reported by the World Health Organization to be approximately 10.1%-15.5% in developing countries, compared with 7.6% in developed countries.

Multiple interventions have been recommended to reduce the risk and complications of CAUTI. The Centers for Disease Control and Prevention (CDC) recommended considering alternatives to indwelling catheters, aseptic techniques for catheter insertion, and reduction of the duration of catheterization [6]. Despite these interventions, CAUTI continues to be a major HCAI in developed and developing countries. A 2016 report showed that in the United States, the incidence of CAUTI was decreasing at a rate that was slower than that of other HCAIs [4]. The International Society for Infectious Diseases (ISID) reported that the frequency of CAUTI was even higher in developing countries (24%) compared to developed countries such as the United States (12.9%) [7].

CAUTI can be caused by bacteria or fungi. Both gram-negative and gram-positive bacteria can cause CAUTI, but the most reported cause is uropathogenic *Escherichia coli* (*E*. *coli*). Antimicrobials are the main therapeutic option for CAUTI. Antimicrobials, unfortunately, have unwanted consequences, including alterations of the normal flora, bacterial resistance, and *Clostridium difficile* infections [1]. These points serve to underscore the importance of the prevention of CAUTI and the dire need for more data that may help reduce the consequences of antimicrobial use.

To our knowledge, there have not been previous studies on CAUTI among critical care patients in Palestine. Therefore, this study aimed to determine the incidence of CAUTI in a Palestinian medical ICU along with the major pathogenic causes, risk factors, and complications of CAUTI.

## Methodology

### Study design, setting, and aim

This study adopted a retrospective observational design, conducted at An Najah National University Hospital (NNUH), a tertiary hospital in Palestine, targeting adult ICU patients who were admitted from January 2018 to November 2023 and who had either indwelling catheters at admission to the ICU or before admission. The study aimed to assess the incidence of CAUTI, as well as the characteristics, pathogens and risk factors for both CAUTI and death.

### Inclusion and exclusion criteria

Patients who were older than 18 years old and admitted to the ICU for 48 hours or longer were included in the study. Patients who had urinary tract infections due to CAUTI or other causes before admission to the ICU *were excluded*.

### Ethics approval and data confidentiality

The proposal for this study was accepted by An-Najah National University Hospital (NNUH). It was also reviewed and accepted by the local International Review Board (IRB) of An-Najah National University. Considering that retrospective data were used, the IRB of An-Najah National University waived the need for informed consent. We ensured strict confidentiality of data throughout our study by anonymizing data to protect individuals’ identities. The data was stored in a secure location to maintain its integrity and privacy.

To make sure our data is accurate and reliable, we took several steps. We cleaned and standardized the data, removed duplicates, and fixed errors. For missing data, we used methods to estimate what was needed. Incomplete records were checked against multiple sources. We also set clear rules for entering data and checked everything regularly.

### Data collection

The data were collected from patient records and nursing flow charts that were accessed after permission from the hospital research committee and started data collection in October 2023. The collected data included the following: demographic data, previous medical history, date of hospital admission and ICU admission, length of hospital and ICU stay, cause of ICU admission, use of certain medications, insertion of any device such as a central venous catheter, antibiotics used before CAUTI, and patient condition in the ICU, whether mechanically ventilated, in shock and type of shock, acute kidney injury, liver failure, or other multiorgan failure. Data related to the urinary catheters that were regularly collected by nurses, including the date of catheter insertion and removal, duration of catheterization, place of insertion in the ICU or ward, and previous history of CAUTI, were collected. For patients who had CAUTI, the identified microorganisms and outcomes after CAUTI including developing septic shock, acute kidney injury, bloodstream infection, and in-hospital mortality, were collected.

### Definitions

CAUTI is defined according to the National Healthcare Safety Network (NHSN) as a UTI that occurred more than two calendar days after an indwelling urinary catheter was placed, with the day of Foley insertion being day one, and the urinary catheter was placed at the time of UTI or a day before. Patients should have at least one of the following: fever more than 38.0°C, urinary frequency, urgency, dysuria, costovertebral angle pain or tenderness, or suprapubic tenderness and should have a positive urine culture >100,000 colony-forming units/millilitre [8].

Immunosuppressive were defined as any use of the following medications: corticosteroids, antimetabolites, calcineurin inhibitors, B-cell depleting agents, or mammalian target of rapamycin inhibitors [9].

### Statistical analysis

Both dependent and independent variables are reported as percentages and means ± standard deviations (SD). Risk factors for CAUTI were assessed by analyzing the association between independent variables (risk factors) and the dependent variable (CAUTI or mortality) using univariate analysis, either an independent t-test for numerical variables or Pearson’s chi-square or Fisher’s exact test for categorical variables. The null hypotheses tested include no association between each independent variable and the risk of CAUTI or mortality. Multivariate analysis was performed to adjust for confounders using binary logistic regression. The specific confounders included in the multivariate analysis using binary logistic regression—sex, diabetes, hypertension, catheterization days, immunosuppressive therapy, steroid use, antifungal medication, coexisting conditions, platelet levels, and kidney injury—were selected based on significant findings from our univariate analysis and supported by relevant literature,

The risk factors for mortality were assessed using the proportional Cox regression model. Allowing for analysis of time-to-event outcomes adjusted for multiple variables. In our study, we calculated CIs around estimates of effect size (e.g., odds ratios from logistic regression) to quantify the uncertainty around these estimates. The p values were set as 2-tailed with an alpha < 0.05. All analyses were performed using the Statistical Package for the Social Sciences (SPSS) Statistics.

## Results

A total of 398 patients were admitted to the hospital’s medical ICU (MICU) during the study period. A total of 377 patients who met the inclusion criteria were analyzed. Of the total patient population, 169 (45%) were female, and 208 (55%) were male, resulting in a male:female ratio of 1:1.2. The most prevalent indication for ICU admission was respiratory failure (37%) (Table 1). Other invasive devices included endotracheal tubes/tracheostomy in 191 patients (51%), central lines in 276 patients (73%), and drains in 70 patients (18%).

**Table 1:** Baseline characteristics and comparisons between patients with and without CAUTI.

Characteristics	Total, n (%) (n = 377)	With CAUTI, n (%) (n = 33)	No CAUTI, n (%) (n = 344)	P-value
**Sex:** Female	169 (45)	21 (63)	148 (57)	0.024
**Age:** year, (mean ± SD)	53.7 ± 18	54 ± 19	53.6 ± 18	0.91
**Comorbidities:**				
Hypertension	168 (45)	21 (60)	147 (42)	0.05
Diabetes	134 (35.5)	13 (39)	121 (35)	0.61
Chronic kidney diseases	73 (19)	8 (24.2)	65 (19)	0.46
Chronic pulmonary diseases	54 (14)	6 (18)	48 (14)	0.51
Cardiovascular diseases	123 (33)	14 (42)	109 (31.8)	0.21
Chronic liver diseases	19 (5)	0 (0)	19 (5.5)	0.39
Malignancy:	137 (36)	14 (42)	123 (35.9)	0.45
Hematological	53 (14)	6 (18)	47 (14)	
Solid	87 (23)	8 (25)	79 (23)	
**Smoking**	104 (27)	8 (24)	96 (27.7)	0.73
**Hospitalization data**				
Cause of ICU admission				
Septic shock	87 (23)	7 (21)	80 (23)	0.78
Sepsis without shock	34 (9)	4 (12)	30 (8.7)	0.5
Respiratory failure	142 (37)	17 (51.5)	125 (36)	0.088
Gastrointestinal bleeding	18 (4)	1 (3)	17 (5)	0.6
Other	93 (15)	4 (11)	89 (26)	
Hospitalization duration, day, (mean ± SD)	20 ± 22.8	53 ± 34	17 ± 18.7	<0.01
ICU duration, day, (mean ± SD)	13 ± 16.7	39 ± 25	10.4 ± 13	<0.01
**Foley related data**				
Catheterization duration, day	10 ± 12.7	32 ± 18	8 ± 9.7	<0.01
**Referral from**				0.79
Ward	153 (41)	12 (36)	141 (41)	
Emergency	63 (17)	5 (15)	58 (17)	
**Laboratory**				
WBC (×109 /L)	14.7 ± 17	16 ± 19.5	14 ± 17	0.69
HGB (g/dl)	9.8 ± 2.7	8.7 ± 1.6	9.9 ± 2.8	0.01
Platelet (×109 /L)	211 ± 169	137 ± 122	217 ± 172	0.009
CRP (mg/L)	119 ± 112	133 ±116	118 ± 112	0.48
Creatinine (mg/dL)	1.8 ± 2.1	1.2 ± 0.7	1.9 ± 2.2	0.08
**Medication**				
Steroid	240 (63)	31 (93)	209 (61)	<0.01
Immunosuppressive	203 (53)	26 (75)	177 (52)	0.003
Aminoglycoside	37 (9.8)	6 (18.2)	31 (9)	0.09
Fluoroquinolone	109 (29)	5 (15)	104 (30)	0.067
Carbapenem	148 (39)	13 (39.4)	135 (39.4)	0.9
Antifungal	90 (24)	14 (42.4)	76 (22)	0.009
Penicillin	83 (12)	7 (21)	76 (22)	0.9
3^rd^ Generation Cephalosporin	46 (12)	2 (6)	22 (12.8)	0.26
Sulfamethoxazole and trimethoprim	32 (8.5)	4 (12)	28 (8.2)	0.44
Vancomycin	161 (42)	17 (51.1)	144 (42)	0.29
Colistin	43 (11)	10 (30.3)	33 (9.6)	<0.01

CAUTI; catheter-associated urinary tract infection, SD; standard deviation, ICU; intensive care unit; WBC; white blood cell, HGB; hemoglobin, CRP; C-reactive protein.

The prevalence of CAUTI was estimated at 9% (33 patients), and the CAUTI incidence per 1000 catheter days was 8.65. Among those who developed CAUTI, 21 (63%) were female, which was a statistically significant on univariate analysis (P-value = 0.024). The mean age of all patients was 53.7 years, with no difference between the two groups (P-value = 0.9). Moreover, smoking history showed no effect on developing CAUTI, as P-value = 0.7, between smokers who had CATUTI and those not. The average number of days of hospitalization for the patient population was 20 days (P-value = <0.01), and the average number of days in the ICU was 13 days (P-value = < 0.01). Moreover, the number of total urinary catheterization days averaged 32 days for CAUTI patients as opposed to 8 days for those who did not develop CAUTI, indicating that longer hospital and ICU stays and longer catheterization days were significantly associated with an increased CAUTI rate (P-value = < 0.01) (Table 1).

Table 1 also shows laboratory investigations on admission to the ICU. Lower hemoglobin and lower platelet counts were associated with a statistically significant increase in the risk of developing CAUTI on univariate analysis. The table also shows several medications that were administered to the patients during the ICU stay before developing CAUTI. Corticosteroids, Immunosuppressive, antifungals, and colistin (polymyxin E) were associated with a significantly greater risk of developing CAUTI, P-value = < 0.01, 0.003, 0.009 <0.01 respectively.

Table 2 shows a comparison between the outcomes that occurred during the ICU stay for patients who developed CAUTI compared to patients who did not. The total mortality rate was 34%. Seventeen (51.1%) of the patients who developed CAUTI died, while 111 (32.4%) of the patients who did not develop CAUTI died; this difference was statistically significant (P = 0.027). Moreover, CAUTI patients were more likely to develop shock, respiratory failure, need for ventilation, bloodstream infection, and acute kidney injury.

**Table 2:** Outcomes comparison between CAUTI and non-CAUTI.

	Total, n (%)	With CAUTI, n (%)	No CAUTI, n (%)	P-value
**Mortality**	128 (34)	17 (51.1)	111 (32.4)	0.027
**ICU condition**				
Shock	213 (56)	25 (75.8)	188 (54.8)	0.02
Septic	195 (50)	23 (69)	162 (47)	
Cardiogenic	4 (1)	1 (3)	4 (1.2)	
Mixed	10 (3)	1 (3)	9 (2.6)	
Respiratory failure	233 (62)	32 (97)	201 (58.6)	<0.01
Mechanical ventilation	211 (56)	30 (91)	181 (52.8)	<0.01
Bloodstream infection	78 (21)	17 (51.5)	61 (17.8)	<0.01
Acute kidney injury	150 (39)	21 (63.3)	128 (37)	0.003

CAUTI; catheter-associated urinary tract infection, ICU; intensive care unit.

**Fig 1:**
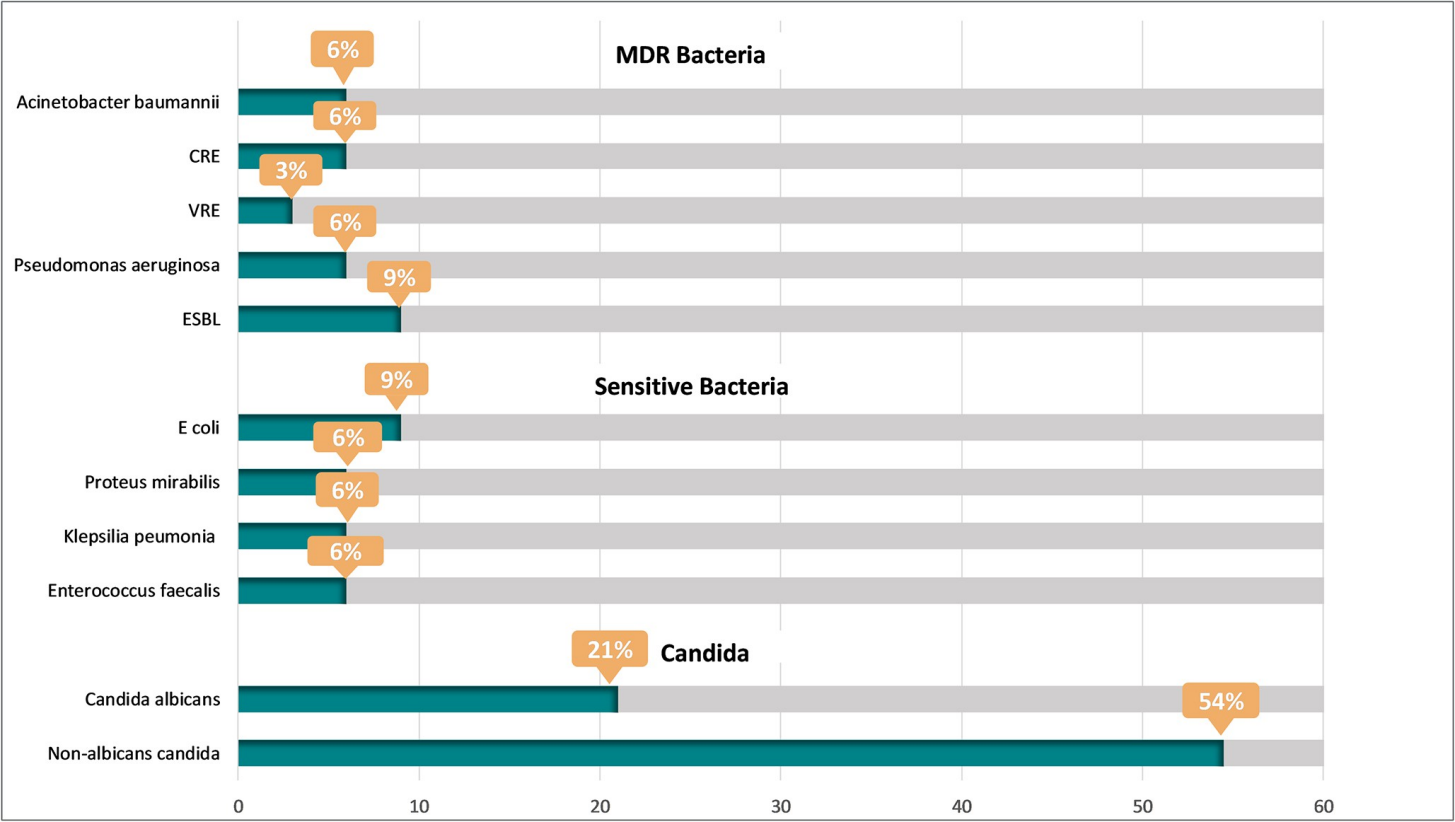
Cultures of isolated organisms from patients with CAUTI. MDR; multidrug-resistant, CRE; carbapenem-resistant Enterobacterales, VRE; vancomycin-resistant enterococci, ESBL; extended-spectrum beta-lactamase, E. coli; Escherichia coli.

The average number of catheterization days before developing CAUTI was 14.2 days. Cultures were polymicrobial in 9 (27%) patients with CAUTI compared to 24 (72%) with monomicrobial cultures, with no statistically significant difference in mortality between the two groups. As shown in Fig 1, most (75%) patients had Candida species isolated from their urine samples. Among all patients with CAUTI, 54% had non-albicans Candida, while 21% had *Candida albicans* isolated in their urine. On the other hand, one-quarter of the patients had bacterial isolates. *Escherichia coli* (*E*. *coli*) was the most commonly isolated bacteria and was found in 9% of all urine cultures. A concerning finding was that 30% of patients had at least one multidrug-resistant (MDR) bacterium isolated. The most prevalent MDR organism in our study were extended-spectrum beta-lactamase (ESBL)-producing bacteria, which were isolated in 9% of the patients.

Factors that showed a statistically significant increase in the risk of developing CAUTI were added to multivariate regression analysis, as shown in Table 3. several variables were found to be significantly associated with the risk of CAUTI. "Sex" (Female vs. Male) demonstrated an odds ratio of 6.8 (95% CI: 1.0–6.8), indicating that females have a significantly higher risk of CAUTI compared to males. Each additional day of "Catheterization" was associated with a 2.5 times higher odds of developing CAUTI (95% CI: 1.2–4.1), underscoring the impact of prolonged catheter use on infection risk. Moreover, "Steroid Use" showed an odds ratio of 3.3 (95% CI: 1.5–5.6), highlighting a notable association between steroid therapy and increased susceptibility to CAUTI.

**Table 3:** Results of logistic regression for the analysis of factors associated with developing catheter-associated urinary tract infection.

Variables		P value	Adjusted OR (95%CI)
	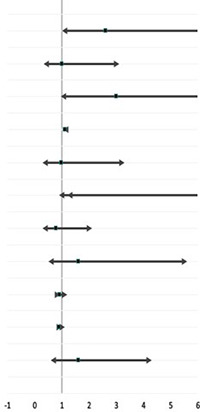		
Sex		0.048	2.6 (1–6.8)
Diabetes		0.98	1 (0.33–3.1)
Hypertension		0.06	3 (0.95–9.4)
Catheterization days		<0.001	1.1 (1.05–1.1)
Immunosuppressive therapy		0.97	0.98 (0.29–3.3)
Steroid		0.03	10.4 (1.2–89)
Antifungal		0.63	0.78 (0.28–2.1)
Colistin		0.39	1.6 (0.51–5.6)
Hemoglobin level		0.5	0.91 (0.93–1.2)
Platelet level		0.12	0.9(0.99–1)
Kidney injury		0.36	1.6 (0.58–4.3)

CAUTI; catheter-associated urinary tract infection, OR; odd ratio, CI; confidence interval.

On the other hand, factors that showed a statistically significant increase in mortality were added to the multivariate regression model, which showed that CAUTI was associated with a significantly increased risk of mortality, with a hazard ratio of 0.4 (0.24–0.77) and a significant P value (0.004). The additional risk factors for mortality were shock, acute kidney injury, and underlying malignancy (Table 4).

**Table 4:** Factors associated with mortality, multivariate regression analysis.

	P value	Hazard ratio (95%CI)
**CAUTI**	0.004	0.4 (0.24–0.77)
Age, year	0.09	1 (0.99–1.02)
Hypertension	0.9	0.97 (0.6–1.6)
Diabetes	0.87	0.96 (0.6–1.5)
Cardiovascular diseases	0.17	0.7 (0.46–1.15)
Malignancy	0.009	1.7 (1.1–2.6)
Shock	<0.001	3 (1.7–5.2)
Respiratory failure	0.27	1.4(0.76–2.6)
Mechanical ventilation	0.47	1.2 (0.7–2)
Bacteremia	0.09	0.7 (0.4–1.1)
Acute kidney injury	0.023	1.6 (1.1–2.3

CAUTI; catheter-associated urinary tract infection, CI; confidence interval.

## Discussion

Our patient population comprised 377 patients with a mean age of 53.7 years, 55% of whom were male. The mean age of patients with CAUTI was 54 years, with a female predominance; 63% of the patients with CAUTI were female. These baseline characteristics are comparable to those of previous studies [10]. The prevalence of CAUTI in our study population was 8.7%, which corresponds to an incidence rate of 8.6 CAUTIs per 1000 catheter days. Previous studies reported widely variable results ranging from 4.8 to 25.4 CAUTIs per 1000 catheter days [11,12]. A study of 21,069 patients admitted to 55 ICUs in 8 developing countries reported an incidence of 8.9 CAUTIs per 1000 catheter days [13]. The same study also reported an overall urinary catheter utilization of 0.73 compared to our slightly greater 0.78 urinary catheterization ratio. Our results are comparable to theirs and are on the lower side compared to other studies, which likely reflects the favorable effect of interventions used in the ICU to reduce the risk of developing CAUTI [10]. The most widely used intervention is the CAUTI care bundle, which aims to avoid using urinary catheters in the absence of an indication and to reduce the number of catheterization days as much as possible.

The most prevalent comorbidities in our patient population were hypertension, diabetes mellitus, cardiovascular disease, and malignancy. None of these comorbidities significantly increased the risk of CAUTI on univariate or multivariate analysis. This contradicts some of the previous studies that reported an increased risk of developing CAUTI with certain comorbidities, especially among those with diabetes [14]. This may be explained by the low event rate in our cohort, which may have caused the analysis to miss such an association. It is imperative to mention, however, that none of these comorbidities has an established firm association with the risk of developing CAUTI.

The most prevalent indication for admission to the ICU in our study was respiratory failure (37%), followed by septic shock (23%), which are among the most frequent indications for ICU admissions worldwide. Though indications for admission to the ICU were not related to the risk of developing CAUTI, the duration of hospitalization and duration of ICU stay were significantly associated with an increased risk of CAUTI. This finding is shared by many nosocomial infections. To date, the most established risk factor for developing CAUTI is the duration of catheterization, which was also evident in our results [2]. Variables related to blood counts, medications administered (except corticosteroids), and kidney injury no longer possessed a significantly increased risk of developing CAUTI following multivariate regression analysis. Though. Although this would naturally mean that the initial association was caused by a confounder, we encountered previous studies that reported that such variables had statistically significant associations [2]. Considering this, we believe that a systematic review or a larger-sized study to test these associations may be the best choice in order to reach a confident conclusion.

On univariate analysis, patients who received steroids, immunosuppressives, antifungals, or colistin (polymyxin E) during admission had a significantly increased risk of developing CAUTI. This significant correlation persisted in multivariate analysis for steroids but not for the other classes. The suppressive effect of corticosteroids on immunity and the consequent increase in the risk of infections are well established [2]. A similar result was reported in a study on stroke patients where CAUTI risk was higher with the use of corticosteroids [15]. Since this does not constitute solid evidence of increased risk, this finding is certainly worthy of further investigation.

Urine cultures were 27% polymicrobial, which is comparable to findings reported by previous studies [16]. Candida species were by far the most prevalent isolates in our study. This contradicts what most centers report on CAUTI. However, a study of 737 health-care-associated UTIs of both adult and pediatric patients in India reported candida as the most common causative pathogen of UTIs in their patient population, causing 29.4% of UTIs [17]. Keeping this in mind, our rate of Candida CAUTI is still much higher than that reported in other studies. Immunosuppressives, which are an established risk factor for fungal infections, were administered to 53% of our patient population (75% of those who developed CAUTI), which might, at least in part, explain this finding. The rising prevalence of non-albicans candida infections is being reported worldwide, and our findings further emphasize this observation [18,19]. As in most previous studies and as is known thus far, the most commonly isolated bacteria in UTIs in general and in CAUTI in particular is *E*. *coli*. In the COMBACTE-MAGNET RESCUING study, which involved 807 complicated UTIs, of which 341 were CAUTIs, *E*. *coli* was the most commonly isolated organism in both groups [16]. A particularly troubling finding was that 30% of CAUTI patients had at least one MDR bacteria isolated. In comparison, a study on complicated UTIs in Bogotá, Colombia, reported that 24.2% of CAUTI isolates produced penicillinases, while 8% were carbapenemase-producing [20].

The overall mortality of our patient population was 128 (34%), where 51.1% of patients with CAUTI died compared to 32.4% of patients with no CAUTI. This increased risk of death was statistically significant with adjustment for other mortality risk factors, and it yielded a crude mortality excess of 18.7, which is comparable to the excess rate reported in the previously mentioned multinational study [13]. The overall high mortality of our cohort is likely related to several factors, the most significant of which are related to the patient. This hospital is a tertiary referral center with a significant proportion of patients having an underlying malignancy (36%), which was found in our study to independently increase the risk of death. Similar to our findings, a study on bacterial CAUTI reported increased mortality among patients who developed septic shock as well as among those who had comorbid cancer. Contrary to our findings, kidney injury had no effect of kidney injury on CAUTI mortality [21].

This study is the first in Palestine to investigate the risk factors, characteristics, and outcomes of CAUTI patients. This study aimed to provide insight into these characteristics and risk factors to help put certain realities in perspective when caring for hospitalized patients, especially those with a urinary catheter. However, it had certain limitations. First, this was a retrospective single-center study, which limits the generalizability of its findings and leaves room for unintentional biases. Second, the relatively low number of CAUTI episodes reported in the study may have led to false-positive or false-negative findings. Third, Lack of data on urinary catheter size and exclusion of patients with a CAUTI diagnosis before ICU admission.

Future research could benefit from prospective multicenter studies to validate our results on a larger scale and explore additional risk factors. Targeted interventions, such as enhanced catheter care protocols and alternative methods to reduce catheterization duration, warrant investigation to mitigate CAUTI rates. Additionally, research focusing on antifungal stewardship and surveillance for multidrug-resistant organisms in CAUTI patients could inform infection control strategies effectively.

## Conclusion

Our study revealed a CAUTI incidence rate of 8.65 episodes per 1000 catheter days. Moreover, female gender, duration of catheterization, and corticosteroid use were associated with an increased risk of developing CAUTI. Fungal pathogens predominated in the urinary cultures in this study. These findings may help formulate antimicrobial management and stewardship plans and emphasize the risk of urinary catheterizations. Finally, despite the frequent attention given to this emerging issue in recent years, it is crucial to strongly emphasize that the problem of resistant bacteria is, to some extent, a consequence of our actions.

## Supporting information

S1 Table Minimal data set. (XLSX)

## Abbreviations

CAUTI: Catheter-associated urinary tract infections
UTI: urinary tract infections
ICU: Intensive care unit
MICU: medical Intensive care unit
HCAI: Healthcare-associated infection
USD: United States dollars
ECDC: European Centre for Disease Prevention and Control
CDC: Centers for Disease Control and Prevention
ISID: International Society for Infectious Diseases
E. coli: Escherichia coli
NNUH: An Najah National University Hospital
NHSN: National Healthcare Safety Network
SPSS: Statistical Package for the Social Sciences
MDR: multidrug-resistant
ESBL: extended spectrum beta-lactamase
CRE: carbapenem-resistant Enterobacterales
VRE: vancomycin-resistant enterococci
OR: odds ratio
CI: confidence interval
SD: standard deviation
WBC: white blood cell
Hgb: hemoglobin
CRP: C-reactive protein
